# Surgery

**Published:** 1895-12

**Authors:** W. H. Harsant


### SURGERY.

The surgical treatment of Graves's disease has been much discussed lately, and a consensus of opinion obtains that much can be done by surgical means for the relief of this affection. One of the most scientific articles on this subject was that by T. Wette,[3] who defines Graves's disease as a reflex neurosis

[3] *Arch. f. klin. Chir.*, 1892, xliv. 652, 764.

of the terminal branches of the sympathetic in the thyroid gland, the exciting agent being the metabolism of the gland. Goitre, in connection with exophthalmos and palpitation of the heart, are the cardinal symptoms for the diagnosis of this disease. This trio of symptoms is ascribed to the effect of a pathological innervation of the sympathetic. The goitre interferes with the normal course of the processes taking place in the gland, producing those alterations which react on the nerve and vessels communicating with it. The sympathetic being known to extend its ramifications to the smooth muscles of the orbit, would produce the exophthalmos. As the same nerve also innervates the fibres of the heart, the palpitation of the heart may be explained in the same manner.

The conservative treatment of exophthalmic goitre, by diet, massage, electricity, application of iodine, &c., having proved at the most palliative, Wette advocates surgical interference as the only treatment indicated. Starting from the hypothesis that the goitre is the *cause* and not the consequence of the alarming symptoms of Graves's disease, he considers the extirpation of the swelling alone capable of producing radical results. But as the total extirpation of the thyroid gland has, in many cases, had very disastrous effects, and since the physiological importance of this gland in the economy of the organism has become manifest, the operations at present are confined to the removal of two-thirds or three-fourths of the thyroid gland, according to its position and connection with the surrounding organs. Riedel has lately improved on the old form of operation by making an arched incision extending from one sterno-mastoid to the other. This has the advantage of the subsequent scar being hidden by the clothing, and of exposing both sides of the swelling at once to the view of the operator. The great danger of these operations is the after-bleeding, which in some cases has proved fatal. Great care, therefore, should be taken in firmly ligaturing the blood-vessels. Another danger is the laceration of the vagus, which has often been enclosed in the goitre, and its normal position altered by the presence of the swelling.

Among the numerous operations for goitre performed by Riedel from July, 1882, to November, 1891, three were for exophthalmic goitre, and were all successful. The following description is given of the most interesting of these cases. Patient a lady, aged forty-nine. The goitre had developed twenty-five years before, and had been treated for several years with iodine, with no other result than the disagreeable effect of the drug. Severe palpitation of the heart, even in repose. Exophthalmos very pronounced. Nervous symptoms, tremor, sleeplessness, alternate heat and cold. The goitre had attained a most extraordinary size, and extended in both directions from the hyoid bone to the clavicle. The sterno-mastoid had been driven from its normal position. The larynx and trachea were entirely covered by the gland. By intra-glandular enucleation, on the right side, a nodule the size of a man's fist was removed. The growth proved parenchymatous, intermixed with fibres and calcareous matter. On the left side and in the centre, extirpation of several smaller nodules followed, while one, which extended under the clavicle, was excised. Before the operation the frequency of the pulse had been 160; directly after, it fell to 84. As soon as the growth was removed the respiration became easy and has remained so, and no recurrence of the palpitation was experienced. The wound healed by first intention, but pleurisy and bronchitis supervened, which kept the patient very weak and retarded recovery. When six months after the operation the patient was seen again she felt perfectly well. The exophthalmos had entirely disappeared. A slight nervousness was the only trouble remaining.

In support of his theory that the enlargement of the thyroid causes the symptoms of Graves's disease, Wette reports two cases from literature, one described by Professor Hack,[1] and another by Professor Hopmann,[2] in which all the symptoms of exophthalmic goitre were proved to be caused by a diseased state of the nose, disappearing when the trouble in the nose had been remedied. He further points out that the atypical symptoms of Graves's disease, the nervous manifestations such as hysteria, hypochondria, epilepsy, tremor, sleeplessness, are also found in cases in which the exophthalmos is wanting, and that these nervous symptoms are caused by the presence of the goitre. These symptoms are at present ascribed to a toxin produced by the pathological thyroid gland which finds its way into the system. In conclusion, Wette sums up as follows:—

1. The principal and surest treatment of Graves's disease is the partial removal of the goitre.

2. The goitre is one of the real causes of Graves's disease:

   *A*. Of its typical symptoms, exophthalmos and palpitation of the heart, either by pressure on the sympathetic or in consequence of the reflex neurosis of this nerve produced by its terminal branches in the thyroid gland.

   *B*. Of the complex of atypical nervous symptoms, the consequence of a general intoxication of the body by chemical metabolism in the pathological thyroid gland.

Professor Mikulicz,[3] of Breslau, says that operative interference in Graves's disease is gaining ground every day, and no one at present doubts the efficacy of this treatment. He has operated on eleven cases; in all of these tachycardia existed in a very marked degree, and five patients presented serious dyspnœa. All survived the operation, which twice consisted in ligature of the thyroid arteries, while in the other nine cases extirpation was practised. Of the patients operated on, six were completely

[1] *Deutsche med. Wchnschr.*, 1886, xii. 425. [2] *Berl. klin. Wchnschr.*, 1888, xxv. 850.

[3] *Med. Week*, 1895, iii. 194.

cured, and in four there was considerable improvement; in one case the improvement was but slight, but in this instance the operation consisted merely in ligature of the thyroid arteries of one side. In the case of one patient, treated by unilateral resection, there was at first great improvement in the morbid symptoms, but the result proved to be ephemeral, for in a short time the tumour on the opposite side developed to such an extent as to compress the trachea. Five months later this side was also extirpated with satisfactory results. The effects of the operation appear much the same, no matter whether the operation be ligature, enucleation, or resection. Operation is by no means free from danger. Although no death occurred in any of his cases, yet he twice met with alarming symptoms, especially rapid pulse and a dyspnœa so intense that he expected to have recourse to tracheotomy. The effects of operation are manifested within a period varying between a few weeks and several months; but whether improvement takes place at an early or remote period, the patients are invariably satisfied with the results, and justly so. It takes a long time for the cardiac disturbances to subside and the exophthalmos to disappear, but this is not surprising. The favourable effect of operation on Graves's disease has been explained in various ways. It cannot be attributed to relief of pressure on the trachea, for dyspnœa is often absent. Nor is the theory of the cervical sympathetic being injuriously influenced by the tumour tenable. It is more likely that there is auto-intoxication by the products of secretion of the hypertrophied thyroid gland, while it is more probable still that the disease begins as a neurosis, and that the symptoms are gradually aggravated by exaggerated secretion of this organ. Professor Kocher,[1] of Berne, considers that the operation of extirpation of goitre in Graves's disease is dangerous, for he has lost three cases; and he has since had recourse to ligature of the thyroid arteries, taking care never to tie more than three of these vessels, because in one case in which he ligated all four arteries the operation was followed by symptoms of cachexia. Professor Krönlein,[2] of Zurich, prefers extirpation of the gland, which he has performed successfully in eight cases. The exophthalmos persisted in one case, but the patient was, nevertheless, well satisfied with the result of the operation. Professor Trendelenburg,[3] of Bonn, has, in some cases of Graves's disease, had recourse to ligature of the thyroid arteries, invariably tying all four, in two sittings, at an interval of a few weeks. He has never met with symptoms of cachexia strumipriva. Professor Rydygier,[4] of Cracow, has practised ligature in twenty-two cases of Graves's disease. He has tied all four arteries without ever noticing any symptoms of cachexia.

Albert H. Freiberg[5] gives statistics of forty-one operations for exophthalmic goitre, with only two deaths; three cases were

[1] *Med. Week*, 1895, iii. 194. [2] *Ibid.* [3] *Ibid.*
[4] *Ibid.*, 195. [5] *Med. News*, 1893, lxiii. 225.

not improved by the operation, but in the other cases, the result varied from complete cure to a more or less partial improvement. For the sake of a better appreciation of the cases he divides them into three classes from the history given. He classes as "surgical" those cases in which the nervous symptoms seemed directly dependent upon the size and growth of the goitre. He considers as "true Graves's disease" only such cases in which the goitre was not the first of the three typical symptoms (tachycardia, goitre, and exophthalmos) to appear, but in which these three symptoms were all present. In the third class, which he describes as "doubtful," he places the remaining cases, where the history reported would not warrant his including them even under such arbitrary heads as the others. Classed as cases of "true Graves's disease" there were then eleven, of which seven were reported cured; and as cases of "surgical Graves's disease" were ten, of which nine were cured; and as "doubtful cases" were twenty, of which nine were cured. If we assume the doubtful cases to have been instances of true Graves's disease, we have thirty-one cases, of which sixteen were cured, that is about fifty per cent. The duration of the cure must greatly interest us. Unfortunately there is nothing mentioned of this in the reports. Only once is there mention of recurrence, and this was not in a case of "true Graves's disease." Three cases are reported as being well five years or more after operation, two after three years, two after two years, three after one and a half years, one after seven months, and one after six months.

James J. Putnam[1] records an operation for Graves's disease done by Dr. J. C. Warren, partly at Dr. Putnam's suggestion, on a patient previously under treatment in the neurological department of the Massachusetts General Hospital. The patient was an unmarried woman, twenty-nine years old. She had never been robust, and had had a number of attacks of protracted vomiting at different times in her life. The "lump in the neck" had been noticed for a year, and the prominence of the eyes and the palpitation were noticed a few months after the enlargement of the neck. When seen, all the symptoms of a moderately severe form of Graves's disease were present—viz., tachycardia, exophthalmos, tremor, nervous agitation, dyspnœa; and besides these, there was an unusual amount of muscular twitching of choreiform character. There was a systolic murmur in the mitral area, transmitted towards the axilla; and the pulmonic second sound was accentuated. In the neck was a soft non-pulsating tumour, descending slightly on swallowing, and this extended from the sternum to the thyroid cartilage, and laterally to the middle of the sterno-mastoid muscle. A loud vascular murmur was heard over the tumour when the stethoscope was applied. Thyroidectomy was performed by Dr. Warren, on February 2nd, 1893. There was a large amount

[1] *J. Nerv. & Ment. Dis.*, 1893, xx. 799.

of bleeding, but no more than is often met with in such cases. After a large plexus of veins had been clamped and tied, the right lobe of the thyroid was shelled out with the fingers, the isthmus clamped, and the right lobe cut off with the scissors. A silk ligature was passed around the isthmus, near the median line, and tied. Then the isthmus was cut off close to the ligature. The pulse and respiration were bad during the operation, and subcutaneous injections of brandy and strychnia were given. During the operation the pulse ran up to 180 and 200, and the prostration was extreme, though there was nothing in the operation itself to cause it. On the afternoon after the operation the pulse was 185, the temperature 104°, and there was great difficulty in breathing. For the next four days she remained in what seemed to be a critical condition, but she gradually improved, and three months afterwards her condition is described as much better than before the operation, but by no means well. The pulse had not dropped below 108, and the patient was anæmic. The gland, after removal, was examined and found to be destitute of colloid. The follicles contained cubical epithelium instead of the normal flat epithelium.

Dr. Putnam points out that there is some liability to sudden death after operation for Graves's disease as well as for other forms of goitre; and this danger is the more alarming because we do not fully understand the cause of the fatal result, nor how to avoid it. The first few days after operation for goitre of any kind are liable to be a distressing period to the patient and an anxious one for the surgeon; but if this period is tided over, a favourable result may be looked for. He gives notes of fifty-one cases where thyroidectomy was performed for Graves's disease, with only four deaths, and in most of the other cases a partial or complete cure resulted; but he points out that these results must not be entirely attributed to the operation, since prolonged rest is always beneficial, and the influence of time is often of itself useful and even curative.

The same author[1] gives notes of two additional cases of thyroidectomy for Graves's disease. The first was a girl, sixteen years old, who had been ill for four years with the signs and symptoms of Graves's disease in a typical form; goitre, exophthalmos, tachycardia, tremor, suffusion of the face, and mental irritability being all present. The right half of the thyroid was removed by Dr. J. C. Warren, on February 2nd, 1894; but although the wound did well, yet restlessness and delirium supervened, and she died a week afterwards, the temperature having risen to 105°, and the pulse to 165. The second case was a young woman, twenty years of age, who was operated on by Dr. Lossander, of Stockholm. Here the result of the operation was excellent at first, but a relapse occurred after two years.

W. H. Harsant.

[1] *J. Nerv. & Ment. Dis.*, 1894, xxi. 359.

